# Differences in the fatty-acid composition of rodent spermatozoa are associated to levels of sperm competition

**DOI:** 10.1242/bio.201411288

**Published:** 2015-03-20

**Authors:** Javier delBarco-Trillo, Rafael Mateo, Eduardo R. S. Roldan

**Affiliations:** 1 Reproductive Ecology and Biology Group, Museo Nacional de Ciencias Naturales, CSIC, 28006 Madrid, Spain; 2 Wildlife Toxicology Group, Instituto de Investigación en Recursos Cinegéticos, CSIC-UCLM-JCCM, 13071 Ciudad Real, Spain; * Present address: School of Natural Sciences and Psychology, Liverpool John Moores University, Liverpool L3 5UA, UK.

**Keywords:** Sperm competition, Sperm membrane, Polyunsaturated fatty acids, PUFA, Arachidonic acid, Lipid peroxidation

## Abstract

Sperm competition is a prevalent phenomenon that drives the evolution of sperm function. High levels of sperm competition lead to increased metabolism to fuel higher sperm velocities. This enhanced metabolism can result in oxidative damage (including lipid peroxidation) and damage to the membrane. We hypothesized that in those species experiencing high levels of sperm competition there are changes in the fatty-acid composition of the sperm membrane that makes the membrane more resistant to oxidative damage. Given that polyunsaturated fatty acids (PUFAs) are the most prone to lipid peroxidation, we predicted that higher sperm competition leads to a reduction in the proportion of sperm PUFAs. In contrast, we predicted that levels of sperm competition should not affect the proportion of PUFAs in somatic cells. To test these predictions, we quantified the fatty-acid composition of sperm, testis and liver cells in four mouse species (genus *Mus*) that differ in their levels of sperm competition. Fatty-acid composition in testis and liver cells was not associated to sperm competition levels. However, in sperm cells, as predicted, an increase in sperm competition levels was associated with an increase in the proportion of saturated fatty-acids (the most resistant to lipid peroxidation) and by a concomitant decrease in the proportion of PUFAs. Two particular fatty acids were most responsible for this pattern (arachidonic acid and palmitic acid). Our findings thus indicate that sperm competition has a pervasive influence in the composition of sperm cells that ultimately may have important effects in sperm function.

## INTRODUCTION

Sperm competition occurs when the spermatozoa of two or more males compete for the fertilization of the same ovum (Parker, 1970; Birkhead and Møller, 1998). Sperm competition is a prevalent phenomenon and its occurrence leads to several evolutionary adaptations both at the morphological and physiological levels (Birkhead and Møller, 1998; Birkhead et al., 2009; delBarco-Trillo et al., 2013). High levels of sperm competition are associated with an increase in the production, storage and allocation of spermatozoa (Parker and Pizzari, 2010; delBarco-Trillo, 2011; delBarco-Trillo et al., 2013), as well as with enhanced sperm function. For example, in rodents, high levels of sperm competition result in a higher proportion of spermatozoa that are morphologically normal, motile, and capable of reaching and fertilizing the ovum (Gomendio et al., 2006; Gómez Montoto et al., 2011b), as well as in modifications in sperm dimensions that may result in improvements in sperm movement (Gomendio and Roldan, 2008; Tourmente et al., 2011). In many taxa, sperm swimming velocity, an important feature of sperm function, is also higher in those species that experience high levels of sperm competition (Fitzpatrick et al., 2009; Kleven et al., 2009; Gómez Montoto et al., 2011a; Tourmente et al., 2011; Lüpold, 2013).

Recently, it has been shown that the increase in sperm swimming velocity observed in rodent species that experience high levels of sperm competition is driven to a great extent by a rise in the content of sperm ATP (Tourmente et al., 2013). That is, in species with high levels of sperm competition there is an increase in metabolism that allows sperm cells to swim faster. This increased metabolism, however, may lead to a rise in the production of reactive oxygen species (ROS). ROS can damage lipids, proteins and DNA of sperm cells. One sperm structure that is especially vulnerable to the oxidative stress caused by increased levels of ROS is the sperm membrane, which is exposed to both intracellular and extracellular ROS. The sperm membrane is involved in sperm motility, sperm viability, and in the processes that precede and enable the fusion of the spermatozoon with the oocyte (Eddy and O'Brien, 1994; Florman and Ducibella, 2006). Lipid peroxidation occurs when lipids react with ROS and can have several negative effects on sperm function, including structural damage to the sperm membrane, loss of motility, and inability to undergo capacitation and fuse with the oocyte (de Lamirande and Gagnon, 1992; White, 1993; Aitken and Bennetts, 2006; Costantini et al., 2010).

As already mentioned, species with high levels of sperm competition experience a generalized enhancement in sperm function (Gómez Montoto et al., 2011b; Lüpold, 2013). Such enhanced sperm function is not compatible with sperm cells suffering oxidative damage. Therefore, any increased production of ROS driven by an increase in metabolism in species with high levels of sperm competition must somehow be counteracted in these species. In principle, there are two main strategies to counteract an increase in ROS: increasing the antioxidant measures that reduce ROS, and changing structures that can be affected by ROS so that they become more resistant to oxidative damage. In the case of the sperm membrane, changes in its fatty-acid composition could diminish the incidence of lipid peroxidation.

The sperm membrane is rich in several types of fatty acids. The proportion of different types of fatty acids can influence many aspects of membrane function (Hulbert and Else, 1999). A key difference among these different types of fatty acids is their level of unsaturation, which is determined by the number of double bonds within the molecule (Wathes et al., 2007). Saturated fatty acids (SFAs), monounsatured fatty acids (MUFAs), and polyunsaturated fatty acids (PUFAs) have zero, one, or more than one double bond, respectively. One of the most polyunsaturated PUFA is docosahexaenoic acid (DHA), with six double bonds. The greater the degree of polyunsaturation of fatty acids, the more susceptible they are to lipid peroxidation (Hulbert et al., 2007). Thus, SFAs and MUFAs are much less susceptible to lipid peroxidation, whereas increasingly unsaturated PUFAs are more prone to lipid peroxidation (Hulbert et al., 2007).

Only one recent comparative study has investigated the relationship that may exist between sperm competition and the composition of the sperm membrane (delBarco-Trillo and Roldan, 2014). That study showed that across mammals high levels of sperm competition are associated with a decrease in n−3 PUFAs, which are the type of PUFAs most prone to peroxidation (delBarco-Trillo and Roldan, 2014). However, that study had two limitations. First, the range of body sizes included in that study was wide, with species ranging from mice to elephants, which introduced a confounding effect because mass-specific metabolic rate (MSMR) is also associated with a decrease in the proportion of n−3 PUFAs in sperm cells (delBarco-Trillo and Roldan, 2014). Second, that comparative study could not carry out an analysis against a control cell type, i.e., a comparison between the fatty-acid composition of sperm cells and somatic cells.

Here we addressed the hypothesis that high levels of sperm competition promote a decrease in PUFA content in the sperm membrane to minimize lipid peroxidation and the ensuing impairment of sperm function. To test this hypothesis we quantified by gas chromatography-mass spectrometry (GC-MS) the fatty-acid composition of different cell types (sperm, testis and liver) in four *Mus* species with similar mass-specific metabolic rates but very different levels of sperm competition (inferred by their relative testes size). We predicted that as sperm competition increases there would be a reduction in the percentage of PUFAs in the membrane of sperm cells. However, we did not expect any relationship between sperm competition levels and fatty-acid composition of testis and liver cell membranes.

## RESULTS

We found that relative testes size (RTS) in the four species followed the pattern that we reported in previous studies, being lowest in *M. pahari*, Thomas (0.24±0.06; range: 0.13–0.29), followed by *M. musculus*, Linnaeus (0.36±0.05; range: 0.27–0.40), *M. spretus*, Lataste (1.19±0.11; range: 1.05–1.34) and *M. spicilegus*, Petényi (1.48±0.06; range: 1.41–1.55; F_3,16_ = 115.3, p<0.0001). All pairwise comparisons (except that between *M. spretus* and *M. spicilegus*) were significantly different. Therefore, if species differences in the percentage of a particular group of fatty acids were to follow a pattern similar to the levels of sperm competition inferred from RTS, then fatty-acid values should be *M. pahari*<*M. musculus*<*M. spretus*<*M. spicilegus* if there is a positive relation (as expected between RTS and % SFAs), or the opposite pattern if the relation is negative (as expected between RTS and % PUFAs).

**Fig. 1.**
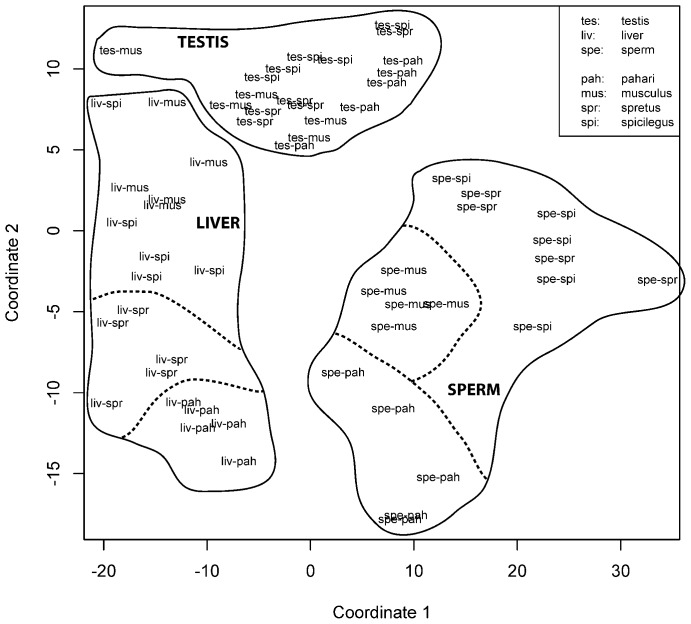
Representation of fatty-acid composition by tissue type and by species. Non-metric multidimensional scaling analysis of fatty-acid composition showing separation of samples by tissue type and partly separation by species within liver samples and sperm samples (*n* = 5 for each species, except *n* = 4 in the case of sperm data for *M. spretus*). Closer distances between any two samples reflect greater similarity in their fatty-acid composition.

The non-metric multidimensional scaling (NMDS) analysis showed a separation of samples by tissue type, i.e. the fatty-acid composition was more similar within samples of one tissue type than between tissue types (Fig. 1). No clear separation by species was observed for testis samples. A separation by species was observed for liver samples, with a clear distinction between samples from *M. pahari* and samples from the other three species. Similarly, a clear separation was observed between sperm samples from *M. pahari* and *M. musculus*, whereas sperm samples from *M. spretus* and *M. spicilegus* were not clearly separated from each other.

A PERMANOVA with the composition of fatty acids as independent variables and species and tissue type as factors indicated that fatty-acid composition differs between species, between tissue types and that there is also a significant interaction between species and tissue type (p<0.0001 in all cases). Post-hoc PERMANOVAs showed that the fatty-acid composition in sperm cells differs from that in liver cells (p<0.0001) and in testis cells (p<0.0001). Additionally, the fatty-acid composition in sperm cells was different between species, except between *M. spretus* and *M. spicilegus* (p = 0.69 between these two species; p<0.01 for all other pairwise comparisons).

**Fig. 2.**
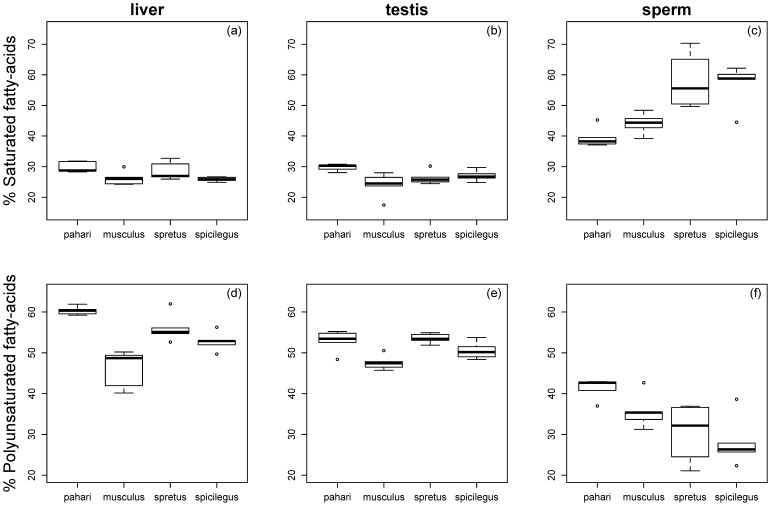
Percentage of saturated and polyunsaturated fatty acids. The percentage of saturated fatty acids (A–C) and polyunsaturated fatty acids (D–F) are shown for liver, testis and sperm. The modified boxplots present the following information: the bar within each box represents the sample median, each box represents 50% of the data around the median, and the two whiskers around each box represent the 95% confidence interval (*n* = 5 for each species, except *n* = 4 in the case of sperm data for *M. spretus*); circles represent outliers.

The % SFAs differed among species in the three tissue types (liver: F_3,16_ = 4.04, p = 0.03, Fig. 2A; testis: F_3,16_ = 4.26, p = 0.02, Fig. 2B; sperm: F_3,15_ = 9.9, p = 0.0007, Fig. 2C). A pattern matching values of RTS in the four species was only apparent in spermatozoa, but not in testis and liver cells, with sperm cells in *M. pahari* (39.49±3.34%) and *M. musculus* (44.07±3.45%) having significantly lower % SFAs than those of *M. spretus* (57.78±9.49%) and *M. spicilegus* (56.84±7.04%; p<0.05 for all pairwise comparisons; Fig. 2C; Table 1). Thus, species with high RTS have higher percentage of SFAs in sperm cells.

**Table 1.**
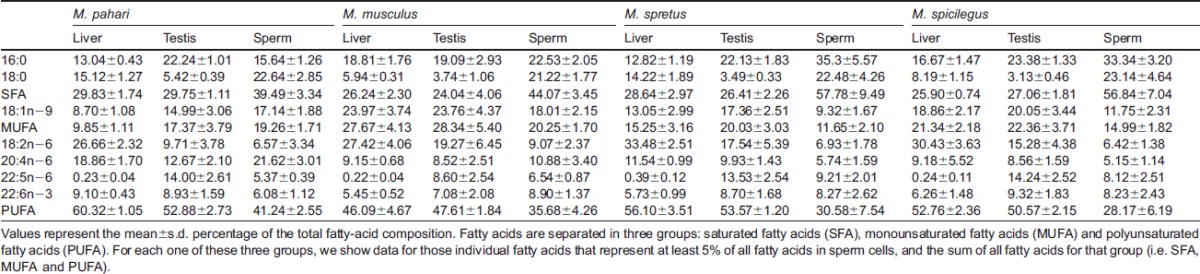
Fatty-acid composition of liver, testis and sperm samples in *Mus* species

The % MUFAs differed among species in the three tissue types (liver: F_3,16_ = 35.1, p<0.0001; testis: F_3,16_ = 6.57, p = 0.004; sperm: F_3,15_ = 21.28, p<0.0001). A pattern that inversely matched values of RTS was only apparent in sperm cells, with *M. pahari* (19.26±1.72%) and *M. musculus* (20.25±1.70%) having higher percentages than *M. spretus* (11.64±2.11%) and *M. spicilegus* (14.99±1.82%; p<0.05 for all pairwise comparisons). That is, species with high RTS had a lower percentage of MUFAs in sperm cells.

The % PUFAs also differed among species in the three tissue types (liver: F_3,16_ = 18.09, p<0.0001, Fig. 2D; testis: F_3,16_ = 4.26, p = 0.001, Fig. 2E; sperm: F_3,15_ = 5.9, p = 0.007, Fig. 2F). A pattern inversely matching values of RTS in the four species was observed only in sperm cells, with decreasing percentages of PUFAs in *M. pahari* (41.24±2.55%), *M. musculus* (35.68±4.26%), *M. spretus* (30.58±7.54%) and *M. spicilegus* (28.17±6.19%). Significant pairwise comparisons, however, were only found between *M. pahari* and *M. spretus* (p = 0.038, Fig. 2F) and between *M. pahari* and *M. spicilegus* (p = 0.007, Fig. 2F). These results reveal a negative association between RTS and the percentage of PUFAs in sperm cells.

**Fig. 3.**
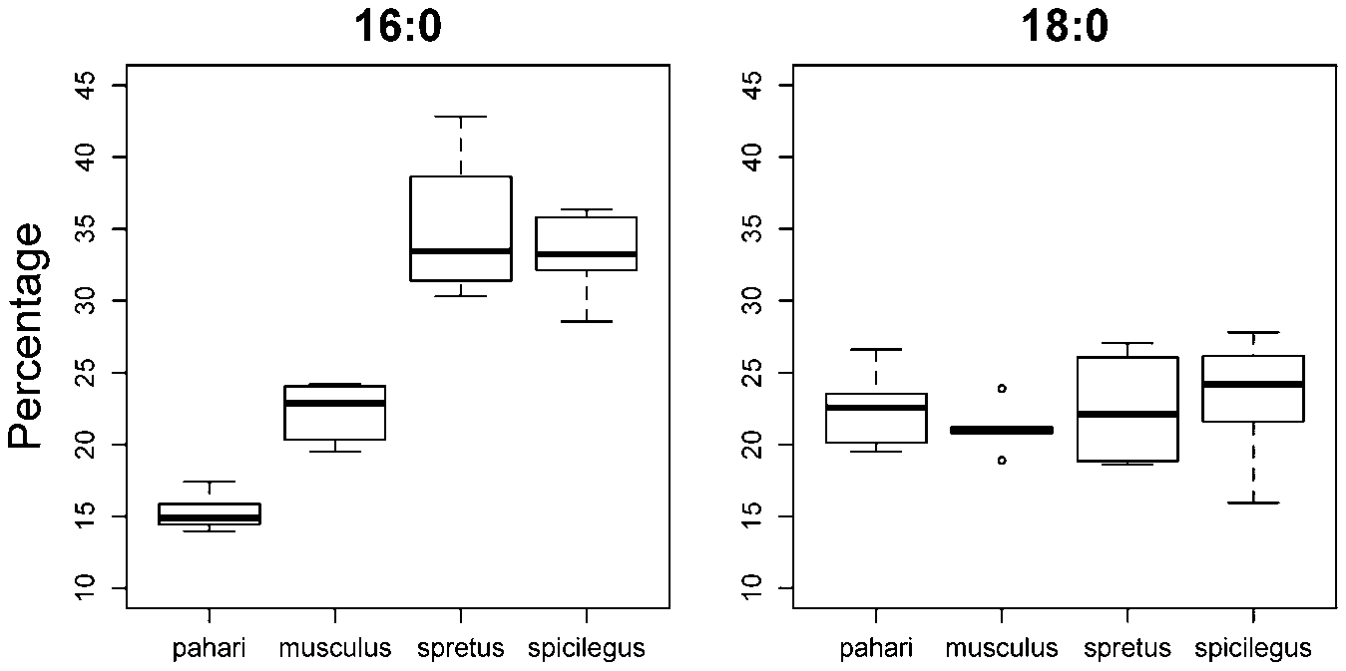
Percentage of the most abundant saturated fatty acids in sperm cells. The two saturated fatty acids that compose more than 5% of the fatty acids in sperm cells are represented. The modified boxplots present the following information: the bar within each box represents the sample median, each box represents 50% of the data around the median, and the two whiskers around each box represent the 95% confidence interval (*n* = 5 for each species, except *n* = 4 in the case of sperm data for *M. spretus*); circles represent outliers.

Only two SFAs (16:0 and 18:0) represented each more than 5% of all fatty acids in sperm cells. Significant differences among species were observed for 16:0 (F_3,15_ = 37.6, p<0.0001) but not for 18:0 (F_3,15_ = 0.29, p = 0.83; Fig. 3). All pairwise comparisons among species for 16:0 were significant (p<0.05) except between *M*. *spretus* and *M. spicilegus* (p = 0.83; Fig. 3). Overall, we observed that the percentage of 16:0 in sperm cells increases as RTS increases.

**Fig. 4.**
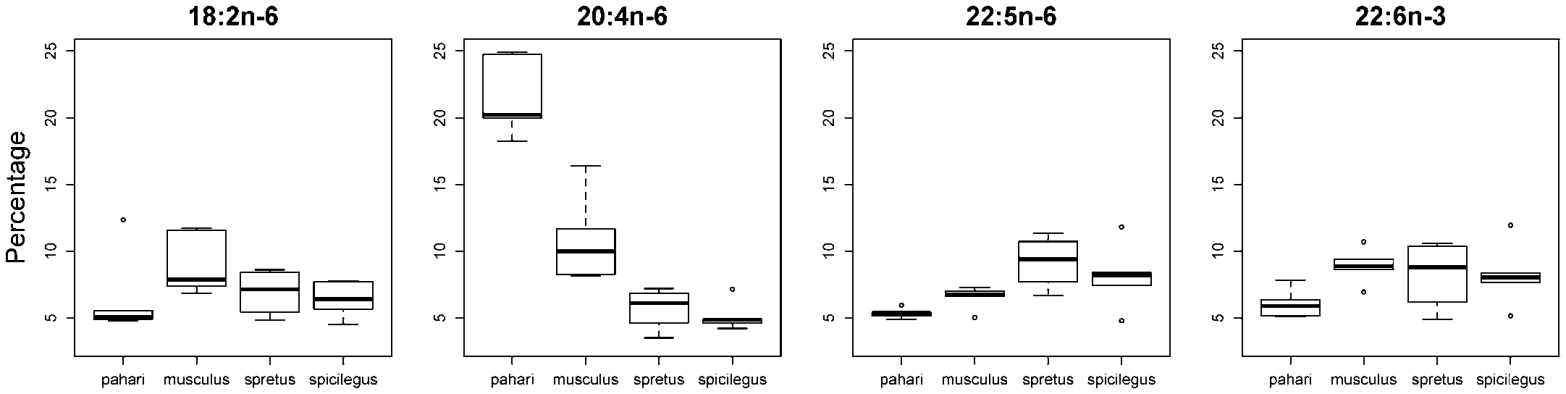
Percentage of the most abundant polyunsaturated fatty acids in sperm cells. The four polyunsaturated fatty acids that compose more than 5% of the fatty acids in sperm cells are represented. The modified boxplots present the following information: the bar within each box represents the sample median, each box represents 50% of the data around the median, and the two whiskers around each box represent the 95% confidence interval (*n* = 5 for each species, except *n* = 4 in the case of sperm data for *M. spretus*); circles represent outliers.

There were four PUFAs that represented more than 5% of all fatty acids in sperm cells: 18:2n−6, 20:4n−6, 22:5n−6 and 22:6n−3. The percentage of two of these fatty acids (18:2n−6 and 22:6n−3) did not differ among species (18:2n−6: F_3,15_ = 1.39, p = 0.28; and 22:6n−3: F_3,15_ = 1.99, p = 0.16; Fig. 4). The abundance of 22:5n−6 differed among species (F_3,15_ = 4.78, p = 0.02) but the only pairwise comparison that was statistically significant was that between *M. pahari* and *M. spretus* (p = 0.02); in any case the differences observed did not match the pattern of RTS among these species. On the other hand, the abundance of 20:4n−6 differed among species (F_3,15_ = 44.11, p<0.0001; Fig. 4) and, in this case, such differences matched the differences among the four species in their RTS. All pairwise comparisons were significant (p<0.05) except that between *M. spretus* and *M. spicilegus* (p = 0.99), indicating that higher RTS is associated with a decrease in the abundance of 20:4n−6.

**Fig. 5.**
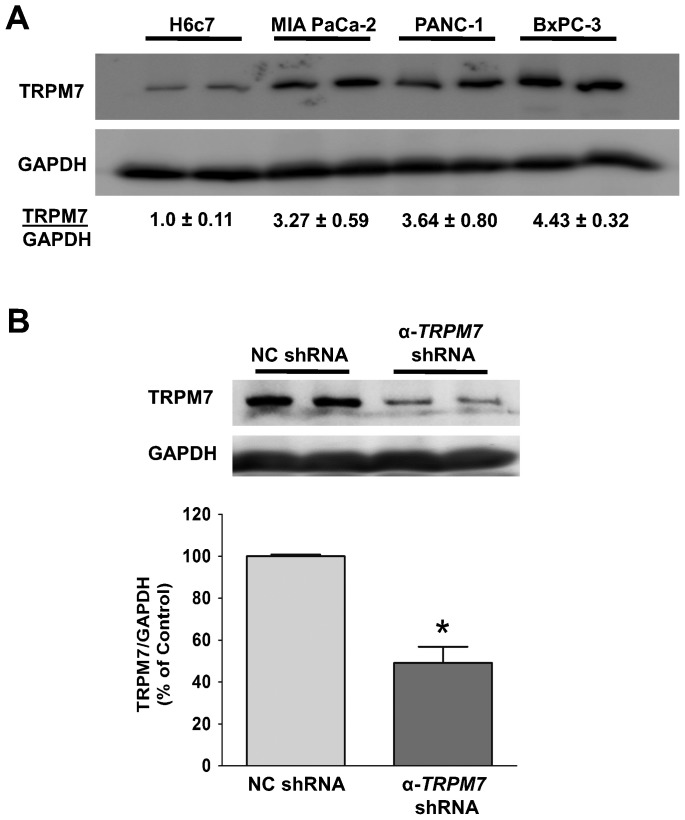
TRPM7 is over-expressed in pancreatic adenocarcinoma cells, and its expression can be down-regulated by shRNA. (A) Cell lysates of each cell line were analyzed for the protein levels of TRPM7 by immunoblotting. The relative amount of TRPM7 and GAPDH protein in each cancer cell line was compared to that in H6c7 as indicated (mean ± standard error). (B) Immunoblotting analysis of TRPM7 protein in BxPC-3 cells transfected with either non-targeting control (NC) shRNA or anti-TRPM7 shRNA. The relative amount of TRPM7 and GAPDH protein in each group of cells is expressed as % of control (NC shRNA). Each column represents the mean ± standard error of three experiments with duplicate samples in each experiment. *indicates statistically significant difference with P<0.05.

**Fig. 6.**
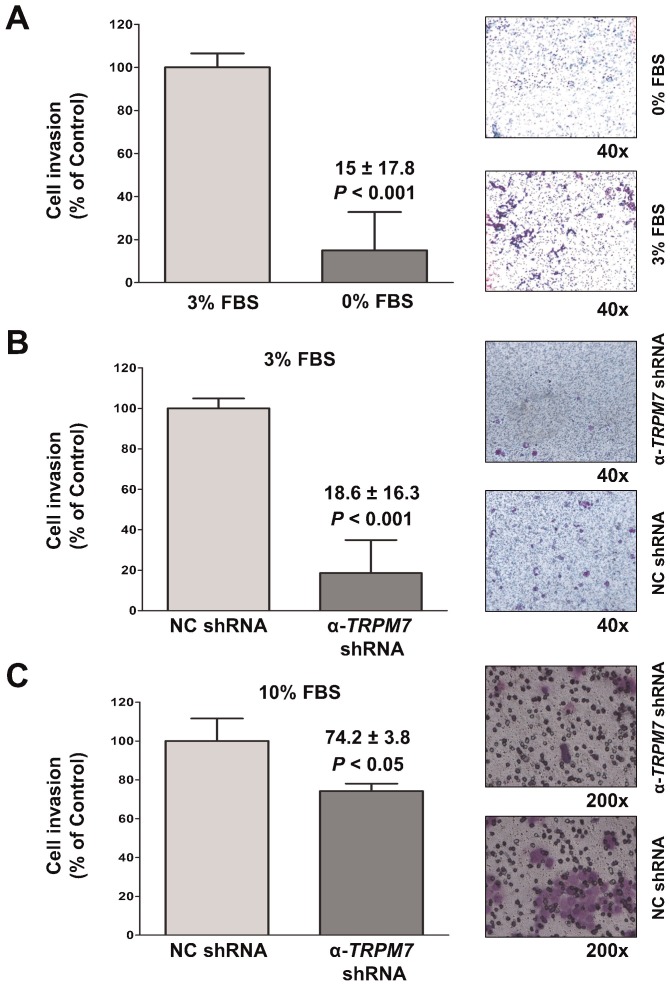
ShRNA-mediated silencing of *TRPM7* impaired invasion in pancreatic adenocarcinoma cells. (A) Non-transfected BxPC-3 cells assayed for invasion using the trans-well assay in the presence of 3% FBS or no FBS. (B,C) BxPC-3 cells transfected with non-targeting control (NC) shRNA or anti-TRPM7 shRNA analyzed for cell invasion in the presence of either 3% FBS (B) or 10% FBS (C). A representative image of the invaded cells stained with crystal violet is shown for each experimental group. Cell invasion is expressed as % control, and each column represents the mean ± standard error.

We found a negative correlation between the percentage of 16:0 and that of 20:4n−6 (partial correlation: estimate = −0.61, p = 0.002).

## DISCUSSION

We found significant differences among four mouse species in the proportion of PUFAs in three types of tissue (sperm, testis, liver). However, as predicted, a pattern matching the inferred levels of sperm competition among these species was only apparent in sperm cells. Specifically, we found that higher levels of sperm competition were associated with a reduction in the percentage of PUFAs in sperm cells. Such a reduction in the percentage of PUFAs was associated with a concomitant increase in the percentage of SFAs. That is, in species with high levels of sperm competition there seems to be a decrease in the percentage of PUFAs associated to an increase in the percentage of SFAs.

It must be borne in mind that the testis tissue analyzed must have included mature spermatozoa within the seminiferous tubules. Consequently, there was the possibility that the results for testis and sperm cells would have shown some overlap, but this was not the case (as shown in Fig. 1). This indicates that the percentage of mature sperm cells relative to the total number of cells in the testis is relatively low, and it also reflects the significant changes in the fatty-acid composition of sperm cells during their transit from the seminiferous tubules to the caudae epididymides (i.e., during epididymal maturation) (Eddy and O'Brien, 1994). We also found that the differences among the four species on the percentage of PUFAs were the lowest in testes cells. In the liver, the percentage of PUFAs was lower in *M. musculus* than in the other three species; we cannot offer at this time a satisfying explanation for this difference.

The decrease in the percentage of total PUFAs and the increase in the percentage of total SFAs associated to high inferred levels of sperm competition was not observed in all individual fatty acids in sperm cells. Instead, among major fatty acids (i.e., those with > 5% of total fatty acids) only one PUFA and only one SFA seemed to be responsible for the overall pattern observed. That is, in species with high levels of sperm competition there was a decrease in the percentage of one PUFA (20:4n−6 = arachidonic acid) accompanied by an increase in the percentage of one SFA (16:0 = palmitic acid). It is not too clear why the decrease in arachidonic acid in species with high levels of sperm competition is balanced by an increase in palmitic acid and not by an increase in the other saturated fatty acid that is also abundant in the sperm membranes of the four mouse species (i.e., 18:0 = stearic acid). In contrast to the positive relationship that we found between the percentage of palmitic acid and levels of sperm competition, the percentage of stearic acid in spermatozoa was very similar in the four mouse species.

Arachidonic acid is a fatty acid prone to peroxidation (Faulks et al., 2006), so its reduction in the sperm membrane of species with high levels of sperm competition should indeed confer some protection against lipid peroxidation in these species. However, following the same argument, we hypothesized that 22:6n−3 (docosahexaenoic acid, DHA) would also be reduced in sperm cells in response to high levels of sperm competition. However, the percentage of DHA in sperm cells did not differ among the four *Mus* species. A lack of relationship between the percentage of DHA in sperm cells and sperm competition levels was also found in a previous comparative study across mammals (delBarco-Trillo and Roldan, 2014). It remains unclear why increased sperm competition does not produce changes in the proportion of DHA, especially when considering that the proportion of DHA in mammalian sperm varies widely across species in comparison with a low variation in somatic tissues. Thus, while the proportion of DHA across species in heart, skeletal muscle, liver, kidney, and brain ranges approximately between 1% and 12% (Hulbert et al., 2002), DHA in sperm cells ranges from very low percentages in rat (0%) and rabbit (1%) to 68% in the African elephant, with a positive association identified between body size and the percentage of DHA (delBarco-Trillo and Roldan, 2014). Our DHA values in sperm cells for *Mus* species (6–9%) matches values previously reported for the laboratory mouse (delBarco-Trillo and Roldan, 2014).

A plausible explanation to why high levels of sperm competition lead to a reduction in the percentage of arachidonic acid but not in the percentage of other PUFAs may be related to the fact that arachidonic acid is a precursor compound (through the activity of lipoxygenases and cyclo-oxygenases) of eicosanoids, including prostaglandins (Joyce et al., 1987; Lax et al., 1990). The oxidation of arachidonic acid into eicosanoids results in the production of both ROS and highly reactive metabolites, which can be relatively long-lived and act not only in the immediate proximity of membranes but can also diffuse from the site of their origin and damage distant targets (Esterbauer et al., 1991; Oborna et al., 2010). Some of these metabolites, for example 4-hydroxy-2-nonenal (HNE) originates not from n−3 PUFAs but by superoxide reaction with n−6 PUFAs, which could partly explain why a reduction in the content of arachidonic acid, instead of DHA, may be a way to prevent an escalation in oxidative damage (Esterbauer et al., 1991). The metabolism of arachidonic acid, and some of the resulting metabolites (e.g. 15-HETE but also other eicosanoids) is also implicated in the acrosome reaction (Fleming and Yanagimachi, 1984; Breitbart and Spungin, 1997). Consequently, a reduction in the percentage of arachidonic acid may reduce the incidence of oxidative stress while also modifying the regulation of the acrosome reaction. For example, a reduction in the percentage of arachidonic acid in species with high levels of sperm competition may lead to a higher integrity of the acrosome, or to a process of acrosomal exocitosis (the so-called acrosome reaction) that is either delayed or whose regulation is more finely tuned and with a different signal threshold. All these acrosomic features should result in a higher percentage of spermatozoa able to achieve the acrosome reaction at the site of fertilization and not during previous stages. In contrast to the seemingly positive effects of reducing the content of arachidonic acid, several intraspecific studies have reported that a decrease in the percentage of arachidonic acid can be associated with a decrease in sperm viability and function: In humans, spermatozoa from asthenozoospermic patients contained lower percentages of arachidonic acid than those of normozoospermic men (Tavilani et al., 2007); in bulls, lower semen quality occurs during the summer, when the contents of arachidonic acid in spermatozoa membranes are also lower (Argov et al., 2007); and epididymal spermatozoa from red deer living in lead mining areas, besides experiencing decreased membrane viability and acrosome integrity (Reglero et al., 2009), contained 50% less arachidonic acid than in control areas (Castellanos et al., 2010).

Sperm cells are normally portrayed as having higher PUFA content and thus being more vulnerable to oxidative damage than other cell types (Mann and Lutwak-Mann, 1981; White, 1993; Aitken, 1997; Sikka, 2001). Such high PUFA content in sperm cells is also thought to confer fluidity to the sperm membrane and be important in the regulation of lipid metabolism and cell movement (Stubbs and Smith, 1984; Gliozzi et al., 2009). This may be the case in large-sized species, such as humans and ungulates, given that a positive relationship exists between body size and the percentage of n−3 PUFAs in sperm cells (delBarco-Trillo and Roldan, 2014), but the present study indicates that in rodents (at least in *Mus* species), sperm cells have lower levels of polyunsaturation than somatic cells, regardless of sperm competition levels. The percentage of PUFAs in sperm cells for *M. pahari* and *M. musculus* is similar to what has been reported in the literature for laboratory *M. musculus* (delBarco-Trillo and Roldan, 2014), whereas the percentage is even lower in *M. spretus* and *M. spicilegus*. The percentage of PUFAs in testis and liver cells, however, are higher than in sperm cells and quite similar in the four species. In conclusion, in *Mus* species sperm cells have generally a low content of PUFAs and high levels of sperm competition can reduce even further the amount of PUFAs in the sperm membrane.

## MATERIALS AND METHODS

### Ethics

The research protocol was approved by the Ethics Committee of the Spanish Research Council (CSIC). All procedures were carried out following Spanish Animal Protection Regulation RD53/2013, which conforms to European Union Regulation 2010/63.

### Animals

We studied adult males (4–6 months of age) from four species of mice from the genus *Mus*: *M. pahari*, *M. musculus*, *M. spretus*, and *M. spicilegus*. These animals come from wild-derived colonies, which have been kept in captivity for only a few generations. Animals were maintained under standard conditions (14 h light–10 h darkness, 22–24°C), with food (rodent chow, Harlan Laboratories) and water provided ad libitum. All males used in this study were housed individually for at least a month before sampling to eliminate the possibility that males had a different perceived risk of sperm competition.

We selected the four aforementioned species because they exhibit very different levels of sperm competition, as revealed by their relative testes size (testes size relative to body mass). Relative testes size (RTS) has been shown to reflect sperm competition levels in rodents (Ramm et al., 2005; Long and Montgomerie, 2006; Bryja et al., 2008; Firman and Simmons, 2008; Soulsbury, 2010). *Mus pahari* has a low RTS, *M. musculus* has a low-intermediate RTS, *M. spretus* has an intermediate-high RTS, and *M. spicilegus* has a high RTS (Gómez Montoto et al., 2011b).

### Obtaining liver, testis and sperm samples

Males (*n* = 5 for each species) were sacrificed by cervical dislocation and weighed. Testes were removed and weighed. One testis and a piece of liver were placed in separate cryovial tubes, snap-frozen in liquid nitrogen and then stored at −80°C. Mature sperm were collected from the caudae epididymides and vasa deferentia by placing the tissue in a Petri dish containing Hepes-buffered modified Tyrode's medium (mT-H; Shi and Roldan, 1995) pre-warmed to 37°C, making several cuts and allowing sperm to swim out for a period of 5 min. We used a hemocytometer to estimate the sperm concentration in the suspension. We then placed a volume of this suspension, containing approximately 9 × 10^6^ spermatozoa, in a cryovial and added mT-H to a final volume of 480 µl. We snap-froze each sperm sample in liquid nitrogen and then stored frozen samples at −80°C until fatty acid methyl esterification and extraction of the corresponding fatty-acid methyl esters (FAMEs). One sperm sample from *M. spretus* was lost after the collection phase, so *n = 4* for sperm data from *M. spretus*.

### Extraction of FAMEs from sperm samples

Immediately after thawing, samples were centrifuged 10 min at 18,620 × *g* at 6°C. We removed the supernatant, added 600 µl of 0.9% NaCl, vortexed and centrifuged again with the same settings. After removing the supernatant, we added 1 ml H_2_SO_4_ to the sperm pellet, vortexed and transferred these contents to a large tube glass containing 70 mg Na_2_SO_4_, and 20 µl of a solution of 0.2 µg tridecanoic acid/µl methanol. The tridecanoic acid (C13:0) was used as an internal standard. To reduce the concentration of oxygen, each tube was placed under a current of nitrogen during 5–10 seconds and closed immediately. Tubes were vortexed and heated at 80°C during 6 hours and then at 60°C overnight. We then added 1.5 ml milliQ water and 0.4 ml hexane to each tube. Tubes were shaken horizontally for 10 min, vortexed and centrifuged. The hexane solution in each tube was transferred to a chromatography vial.

### Extraction of FAMEs from liver and testis samples

After weighing a piece of the organ, we ground it in a mortar with a ratio of 9 parts Na_2_SO_4_:1 part organ. We placed the resulting content in a large glass tube together with 20 µl of a solution of 10 µg tridecanoic acid/µl methanol and 3 ml H_2_SO_4_. Each tube was placed under a current of nitrogen during 5–10 seconds and closed immediately. Tubes were vortexed and heated at 80°C during 6 hours and then at 60°C overnight. We then added 3 ml milliQ water and 1.8 ml hexane to each tube. Tubes were shaken horizontally for 10 min, vortexed and centrifuged. Each hexane solution was transferred to a chromatography vial.

### Identification and quantification of FAMEs

We injected 1 µl of each FAME solution in an Agilent Technologies 6890N GC Network System with a 7683 series injector and coupled to an electronic impact-mass spectrometry detector (EI-MS; 5973N MSD; Agilent Technologies). The capillary column used was a BPX70 column (30 m × 0.25 mm i.d., 0.25 µm film thickness; SGE Analytical Science). The injector in split mode (20:1) was set at a temperature of 270°C and the oven was maintained for 5 min at 100°C after injection and then increased to 240°C at a ramp rate of 2.5°C/min. The carrier gas was helium at a flow rate of 1.2 ml/min. The source inlet of the mass spectrometer was held at 230°C and 70 V. The identification and quantification of FAMEs was achieved by comparison to retention times of FAME reference standards (FAMQ-005, AccuStandard, New Haven, USA) and other FAMEs identified before in liver, testis and sperm samples with the same analytical method (Reglero et al., 2009; Castellanos et al., 2010), and by their mass spectra. Calibration curves were performed with FAMEs concentrations ranging from 0.06 ng/µl to 11.89 ng/µl. Blanks were processed with each batch of samples.

### Statistical analyses

All statistical analyses were conducted using R version 2.15.2 (R Core Team, 2012). Percentage data were arcsine transformed (calculating arcsine of the square root of the variable) prior to analyses. Average values are reported as mean±s.d. Significance level (α) was set at 0.05 for all the tests.

To test whether the relative testes size (RTS) of the animals that we sampled followed the predicted pattern (i.e., lowest RTS in *M. pahari* and then progressively higher in *M. musculus*, *M. spretus* and *M. spicilegus*), we carried out an ANOVA to determine if RTS differed among species. The RTS values were calculated using the formula RTS = testes mass/0.031 × body mass^0.77^ (Kenagy and Trombulak, 1986).

To obtain an initial assessment of the similarities among samples depending on tissue type and species, we reduced the multidimensionality of the fatty-acid information to two arbitrary dimensions, the location of samples in the resulting two-dimensional space representing their fatty-acid composition, with closer distances between two samples reflecting greater similarity in their fatty-acid composition. Principal component analysis was not an appropriate technique because we had a sparse matrix (i.e. there were many zeros) that did not approximate multivariate normality despite several transformation attempts (McCune and Grace, 2002). The most appropriate technique was non-metric multidimensional scaling (NMDS), which is robust to a large number of zeros and does not assume multivariate normality (McCune and Grace, 2002; Zuur et al., 2007). We implemented a Kruskal NMDS using the R function isoMDS in the MASS package and selecting two dimensions. Given that NMDS does not offer any formal statistical test, we also conducted PERMANOVAs (using the function adonis in R) to determine if the compositional differences in fatty acids differed significantly between species and between tissue types.

For the fatty-acid variables under study (see below), we carried out an ANOVA for each tissue type. Normality was checked with the Shapiro-Wilk test and homogeneity of variances with the Bartlett test. When necessary, pairwise comparisons were conducted using the Tukey Honest Significant Difference method (using the function TukeyHSD in R). We considered two types of fatty-acid variables. First, we used three variables that summarized a subset of fatty acids: % SFA, % MUFA, and % PUFA. Second, we considered those SFAs and PUFAs that represented at least 5% of the total of fatty acids in sperm cells. After detecting significant differences among species in the proportion of one SFA (16:0) and one PUFA (20:4n−6), we performed a partial correlation (using the function pcor.test in R) to determine if there was a significant relationship between the proportions of these two fatty acids across samples while controlling for species.

### List of abbreviations

DHA, docosahexaenoic acid; EI-MS, electronic impact-mass spectrometry detector; FAME, fatty-acid methyl ester; GC-MS, gas chromatography-mass spectrometry; HNE, 4-hydroxy-2-nonenal; mT-H, Hepes-buffered modified Tyrode's medium; MSMR, mass-specific metabolic rate; MUFA, monounsatured fatty acid; NMDS, non-metric multidimensional scaling; PUFA, polyunsaturated fatty acid; ROS, reactive oxygen species; RTS, relative testes size; SFA, saturated fatty acid.
